# Supplementary material for: Detecting anthropogenically induced changes in extreme and seasonal evapotranspiration observations
Source: Nat Commun. 2026 Jan 23;17:879. doi: 10.1038/s41467-025-67748-8 (PMC12830918; doi:10.1038/s41467-025-67748-8)
Supplement: Supplementary file 1 — Supplementary Information [file 41467_2025_67748_MOESM1_ESM.pdf]

Detecting Anthropogenically Induced Changes in Extreme and Seasonal Evapotranspiration Observations: Supplementary Information

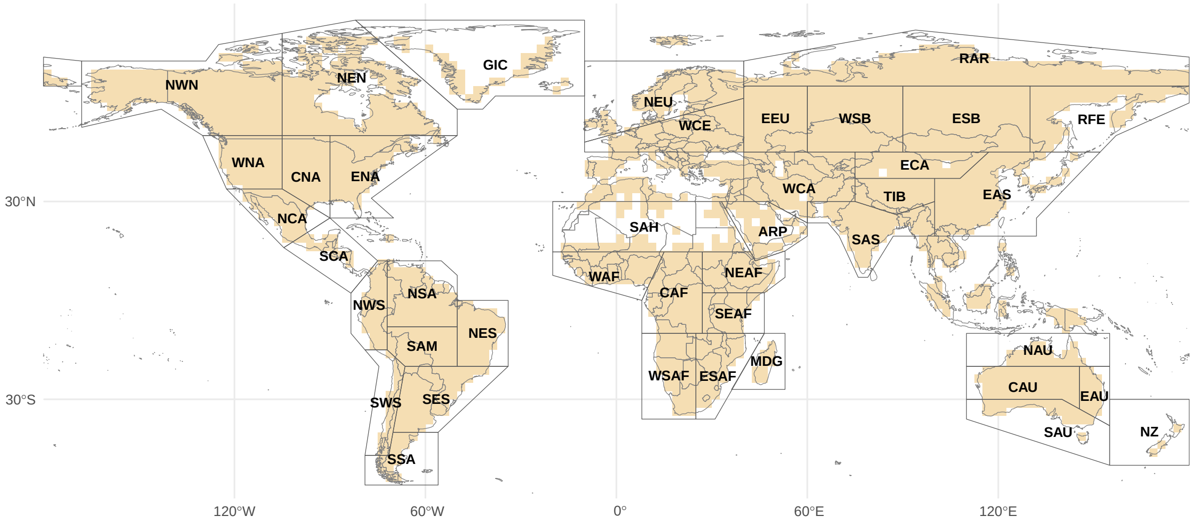

Supplementary Figure 1: SREX regions used in the study. The coloring indicates the mask which was used for all data. Map made with Natural Earth.

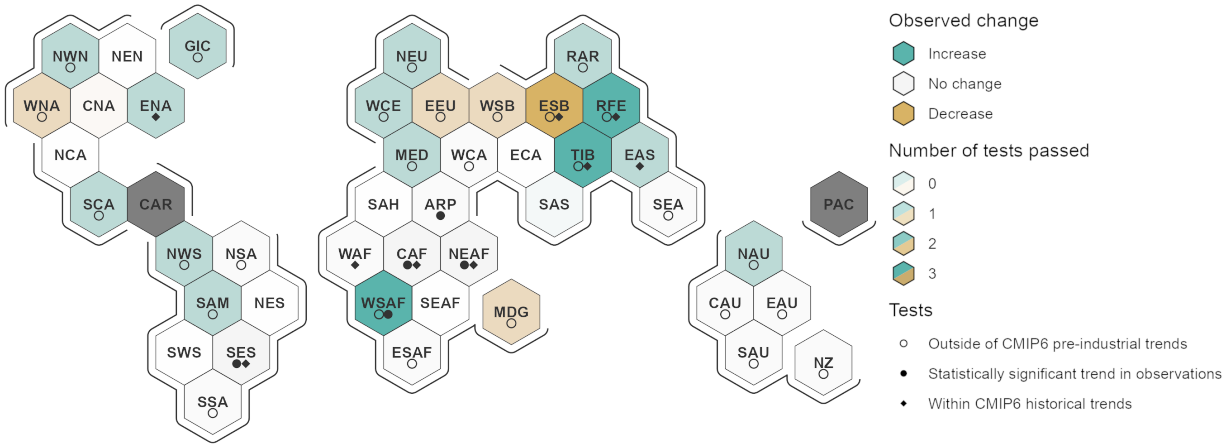

Supplementary Figure 2: ETx7d (a) and JJA ET (b) trends in SREX regions over land. The color indicates regions where ERA5 Land and GLEAM agree on the sign of the 1980–2023 trend. The strength of the color indicates the number of the three tests a region passes. When these two trends lie within the distribution of CMIP6 historical trends, a circle is added to the region. When both observational trends lie outside the CMIP6 pre-industrial distribution, the black dot is added. Finally, when the observed trends both pass a Mann-Kendall significance test, the diamond is added.

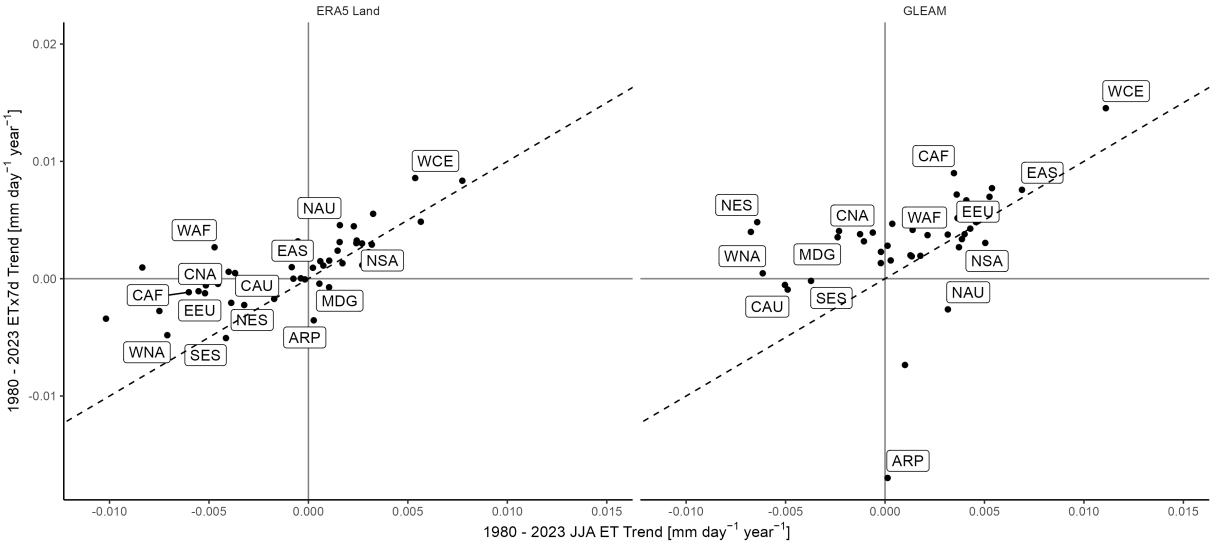

Supplementary Figure 3: Observed regional mean JJA ET trends vs ETx7d trends. Every dot represents a SREX region. The dashed line has a slope of 1 and an intercept of 0, to give visual support for comparing the sizes of the ETx7d and the JJA ET trends. A selection of SREX regions has been labeled.

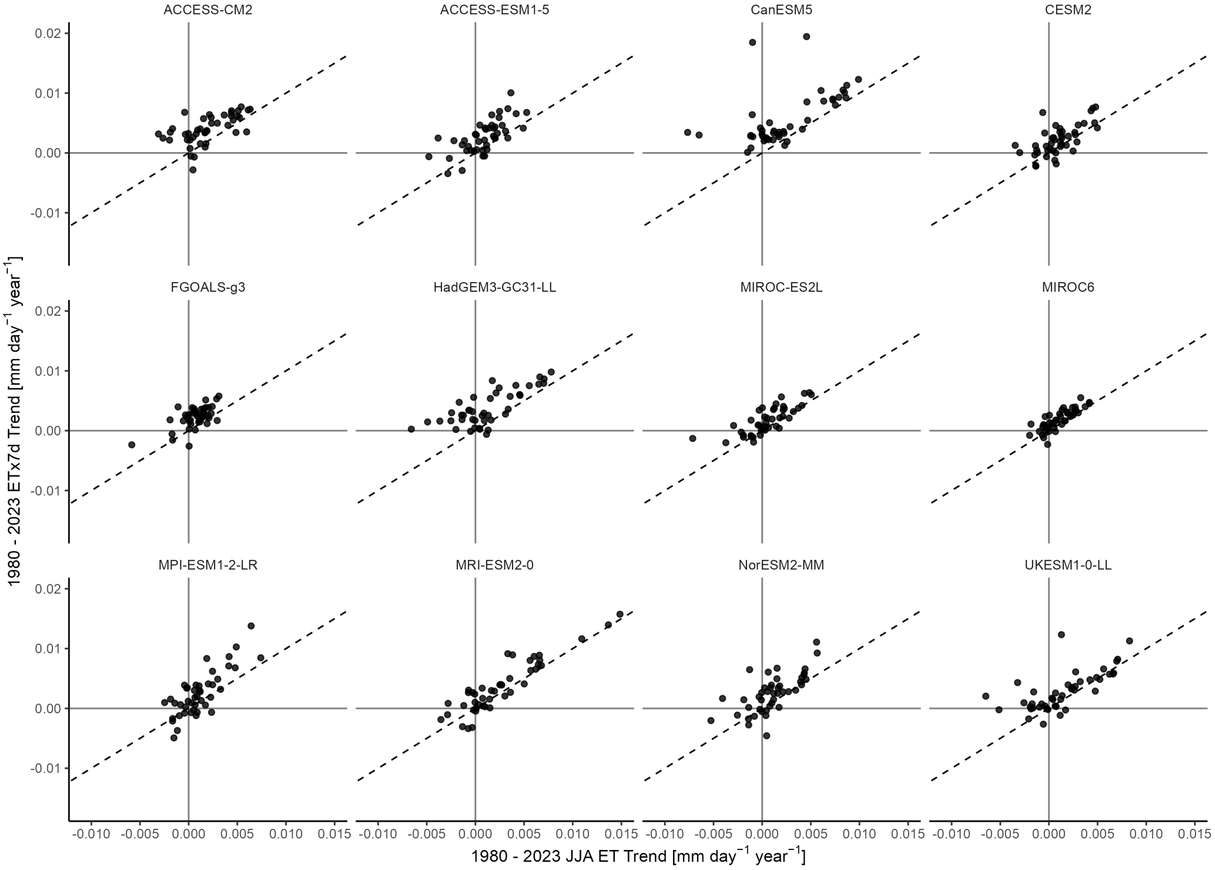

Supplementary Figure 4: CMIP6 model mean historical and SSP5.85 regional mean JJA ET trends vs ETx7d trends. Every dot represents a SREX region. The dashed line has a slope of 1 and an intercept of 0, to give visual support for comparing the sizes of the ETx7d and the JJA ET trends.

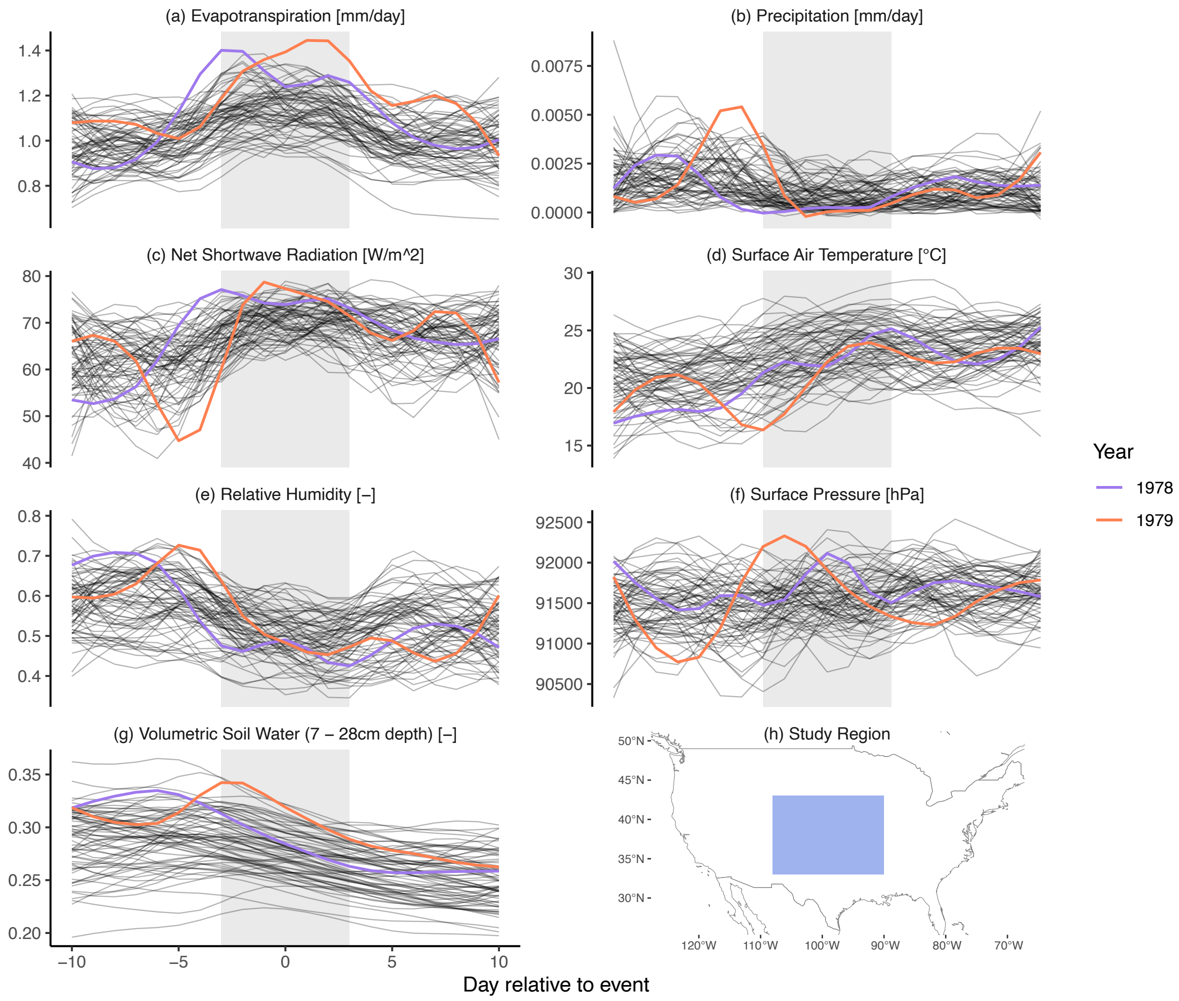

Supplementary Figure 5: Composite time series of various variables during an extreme ET event in ERA5 Land. The x-axis indicates days relative to the event with 0 being the center of the 7-day event. The gray box indicates the 7-day duration based on which the events were selected. Two lines are colored and indicate the strongest (1978) and second strongest (1979) events in the study region (h), as well as a year with a substantial flash drought in 2012. The other lines indicate the evolutions of all other events between 1950 and 2023. A loess smoother was applied to improve visual clarity.

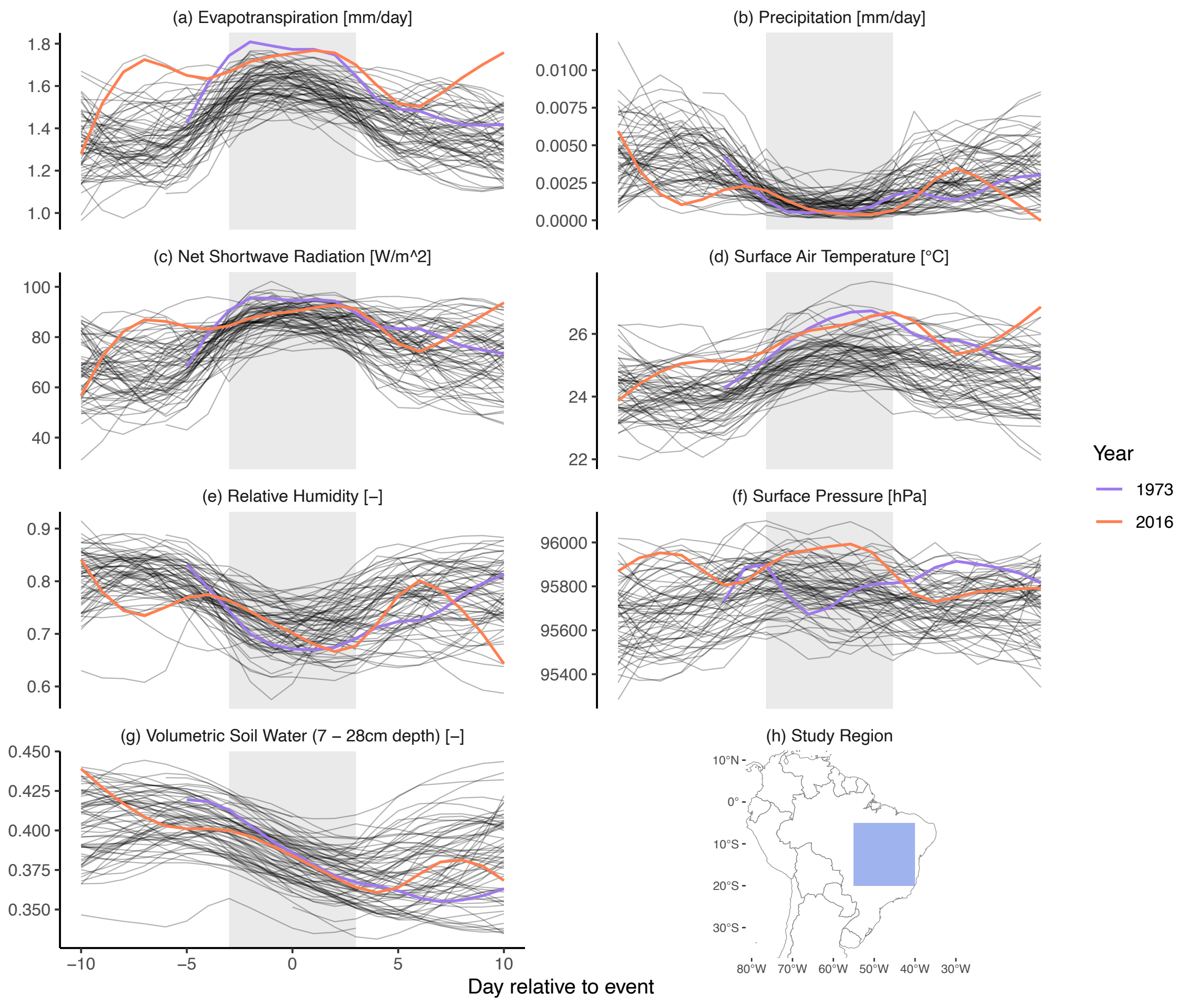

Supplementary Figure 6: Composite time series of various variables during an extreme ET event in ERA5 Land. The x-axis indicates days relative to the event with 0 being the center of the 7-day event. The gray box indicates the 7-day duration based on which the events were selected. Two lines are colored and indicate the strongest (1973) and second strongest (2016) events in the study region (h). The other lines indicate the evolutions of all other events between 1950 and 2023. A loess smoother was applied to improve visual clarity.

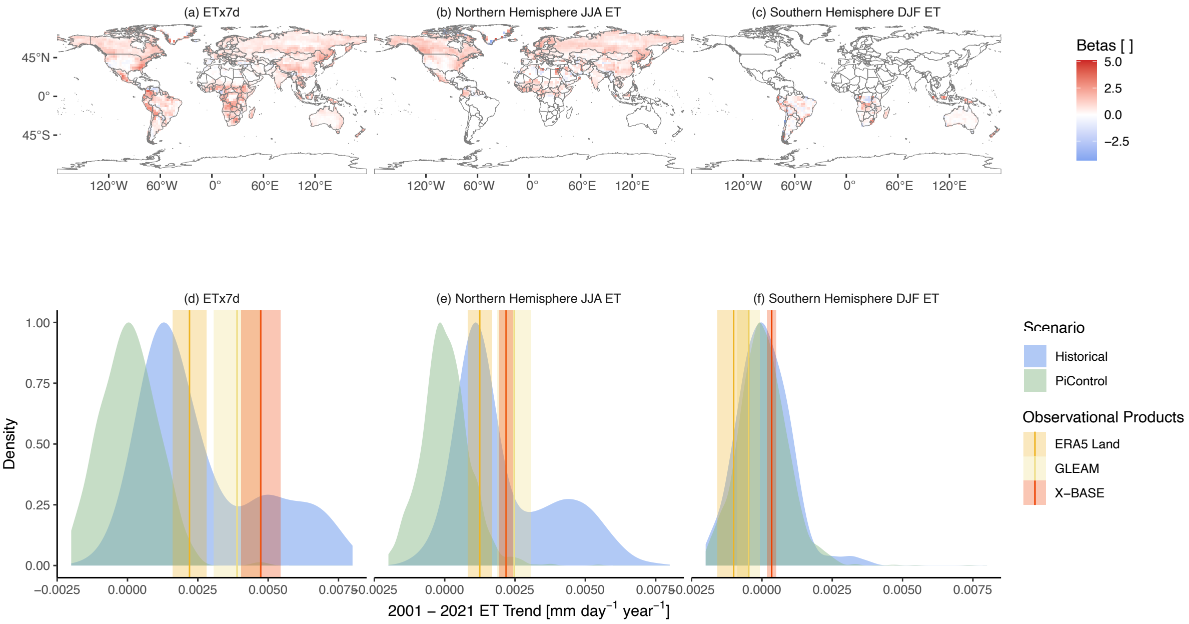

Supplementary Figure 7: Mapped coefficients for predicting the forced response of global mean ETx7d (a), Northern- (b), and Southern (c) hemisphere summer mean ET. The coefficients have been scaled to the same variance but not centered, so positive values indicate positive correlation with the forced response. ETx7d (d) , Northern hemisphere JJA ET (e), and Southern hemisphere DJF ET (f) forced trend density of historical (blue) and PiControl (green) climate model simulations between 2001 and 2021. Observational trend of GLEAM, ERA5 Land, and X-BASE are indicated by the vertical lines, as well as the bootstrapped standard deviation by the shading around the lines. Maps made with Natural Earth.

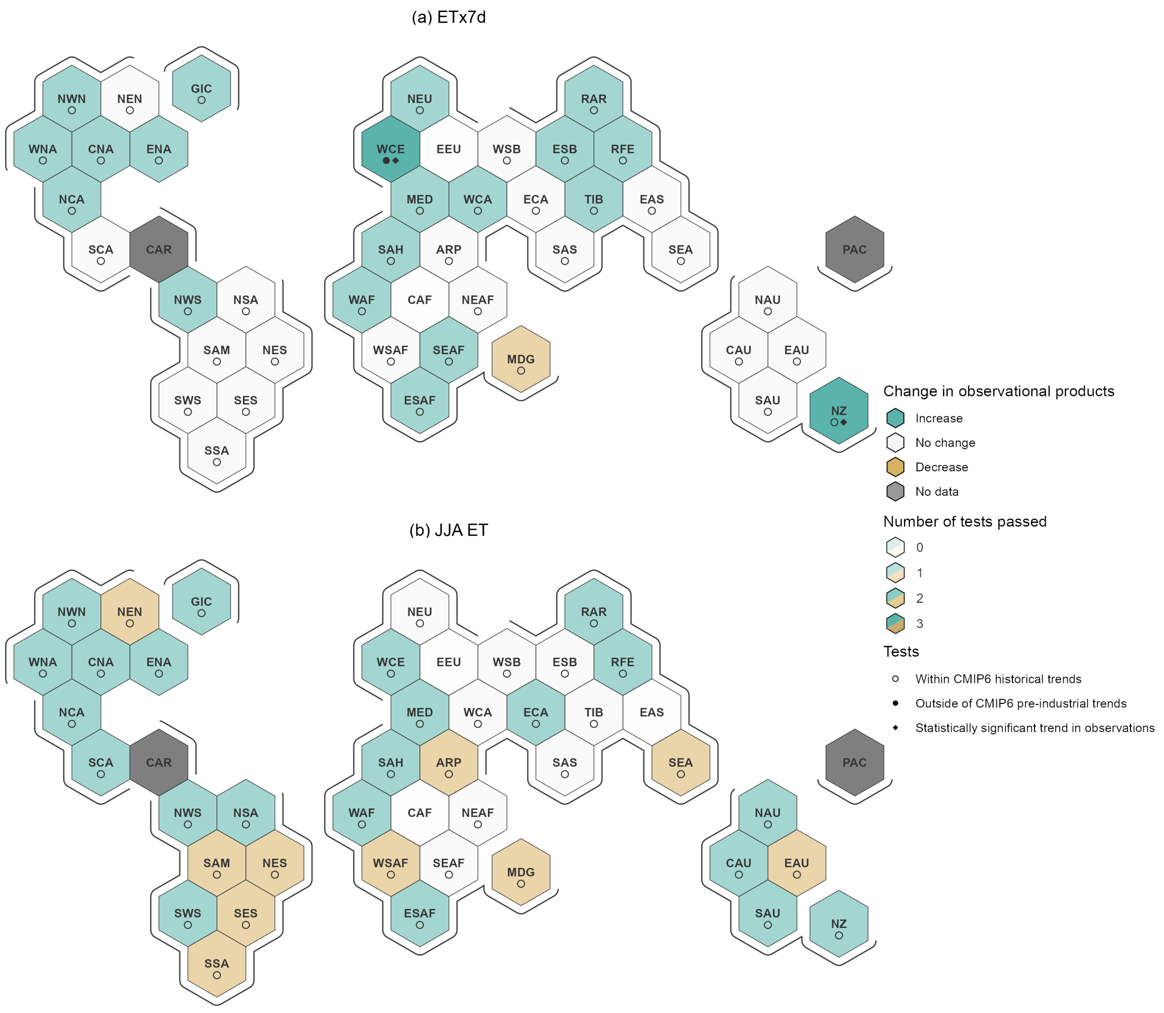

Supplementary Figure 8: ETx7d (a) and JJA ET (b) trends in SREX regions over land. The color indicates regions where ERA5 Land, GLEAM, and X-BASE agree on the sign of the 2001–2021 trend. The strength of the color indicates the number of the three tests a region passes. When these two trends lie within the distribution of CMIP6 historical trends, a circle is added to the region. When both observational trends lie outside the CMIP6 pre-industrial distribution, the black dot is added. Finally, when the observed trends both pass a Mann-Kendall significance test, the diamond is added.

CMIP6 Model	ETx7d members	ETx7d PiControl years	Seasonal ET members	Seasonal ET PiControl years	Group for cross-validation
ACCESS-CM2	9		5	500	A
ACCESS-ESM1-5	30		40	1000	A
CanESM5	50		50	2051	B
CESM2	2	1200	5	1200	C
CESM2-WACCM	3	1082	510		C
CMCC-CM2-SR5		250			
CMCC-ESM2		250			
CNRM-CM6-1			6	500	D
CNRM-ESM2-1			5	500	D
EC-Earth3			8	501	E
EC-Earth3-Veg			8	2000	E
HadGEM3-GC31-LL	3	300	4		A
HadGEM3-GC31-MM	4	250			A
IITM-ESM		175			
IPSL-CM6A-LR			7	2250	F
MIROC-ES2L	9	250	10	500	G
MIROC6	50		48	800	G
MPI-ESM1-2-HR	2				H
MPI-ESM1-2-LR	30		30	1100	H
MRI-ESM2-0	5		6	952	J
NorESM2-LM	1	501	1	500	C
NorESM2-MM	1	500	1		C
TaiESM1		500			
UKESM1-0-LL	6	190	5	1880	A

Supplementary Table 1: CMIP6 models, number historical and SSP5-8.5 ensemble members and the number of PiControl years used to obtain the Null distribution. CMIP6 models and number of members differ based on availability. The last row shows the CMIP6 model-group that was used to cross-validate the Ridge regression model.
